# Remarks on Some Forms of Abdominal Neuralgia Depending on Uterine Irritation

**Published:** 1843-11

**Authors:** Golding Bird


*Remarks on some forms of Abdominal Neuralgia depending on Uterine Irritation.* By Golding Bird, A.M., M.D., F.L.S., &c.—[It seems to be more and more the opinion of the profession that we are only half acquainted with those "protean host of maladies or rather symptoms simulating every form of inflammatory affection occurring in women, from the period of the first developement of the uterine functions to the time of their cessation." How many women have been bled and blistered for pleuritis and other supposed inflammatory affections, or confined for months on couches, and gone through the ordeal of blisters, leeches, and moxa, when the uterus was the real organ affected, and clearly produced those distant neuralgic symptoms. When we know how the unimpregnated womb, when in a state of irritation, exercises its influence on distant organs, we need not be surprised that this is much more the case when that organ has been lately undergoing immense changes from impregnation; and also when delivery has been accomplished, and it is gradually re-assuming its former size and functions. "Accordingly, at a time varying from a few hours to a few weeks after parturition or miscarriage, we occasionally find excessive irritation of the uterus, attended with intense neuralgic pain over the abdomen, and a suppression of the lochial discharge manifested, producing a disordered state of every function of the body, giving origin to a set of symptoms often mistaken for puerperal fever and peritonitis." Dr. Bird relates a good case of this kind. Susan Haws, aged 29, was admitted under his care at the Finsbury Dispensary. She had always been ailing since menstruation commenced, and worse since marriage; she had pains in the hip and loins as well as over the pubes, darting to the back and extending to the left breast and between the shoulders, with excessive low spirits and fits of an hysteric character.]

"All these symptoms are always much more intense when the catamenia appear. For the last two years she has been troubled with excessive leucorrhœal discharge. Two days before she became my patient, she was seized with a fit of an epileptic character, she being then at the termination of a third pregnancy. For this she was bled copiously, and a few hours aftewards was delivered of a healthy child. During the last few months of utero-gestation, she had been subject to diarrhœa, and the genital organs had been so irritable, that sexual intercourse was attended with excessive pain, and was always followed by an increase of the leucorrhœal discharge, and of the pains above described. After her labor, the lochiæ became copious, her milk abundant, and she appeared well until forty-eight hours had elapsed, when she came under my care for an attack, as it was stated, of peritonitis. I found her lying in bed on her back, with her legs drawn up; her abdomen tumid, and so exquisitely tender that the slightest touch produced the most intense pain; her features were contracted, and presented an aspect of the utmost anxiety; her eyes were suffused; her tongue white, but moist; her pulse 100, small, but jerking; the urine was very scanty, and nearly brown from its extreme concentration. The woman did not lie still and quiet, but kept tossing about from side to side uttering loud and incoherent screams; she threw her arms wildly over her head, and seemed scarcely conscious of any thing around her, until an attempt was made to touch, *or even approach* the abdomen, when she shrunk back and cried out in an incoherent manner. I found that she had not slept the whole night from excessive rigors, which had continued nearly without intermission for eight hours, and that these were followed by heat and sweating and had terminated in the attack

I witnessed. The abdominal pain appeared to be most intense about the pubic region and over the cœcum, and to become more severe every five or ten minutes, never however entirely ceasing.

The practitioner who had been called to this patient early in the morning before I saw her, had covered the abdomen with leeches, as her friends resolutely refused allowing her to be bled from the arm, and had given a large dose of calomel; the pain had, however, become much more severe, and the paroxysms more frequent since its administration. The lochiæ had ceased in the course of the night, and the bowels had been once opened during the previous twenty-four hours.

℞. Hydrarg. c. Creta. gr. iv. statim; Olei Ricini. ℥vj. post horam; Opii puri, gr. j.

Antim. Tart. gr. 4, Misce. Ft. pilul, nocte maneque cap. And a large bran-bag fomentation was ordered to be applied to the abdomen as hot as it could be borne, and continued during the day. She was ordered to take mutton-broth and gruel *ad libitum*.

Feb. 19. A scanty show of the lochiæ has appeared in the night; bowels freely open; dejections dark, fœtid, and containing scybalæ; she had had some sleep in the night; tongue white but moist; pulse 100, small and compressible; abdomen exquisitely tender on pressure, but the paroxysms of pain are less frequent, although still severe; she still utters loud cries on the slightest pressure being applied to the abdomen; she has passed no water, and not above an ounce of turbid, dark urine was removed by the catheter; the surface is still hot, but much moister; the bran-bags have been kept constantly applied. Rep. Ol. Ricini, pilulæ, et fotus.

20th. Has passed an excellent night; bowels freely open, with much dark, fœtid, and somewhat scybalous matter; no fresh rigors; lochiæ copious; abdominal tenderness less; the paroxysms of pain have nearly ceased; urine abundant. Rep. pilulæ et fotus.

She continued mending until the 25th, when she was reported convalescent; still, however, having an irritable condition of the uterus left, as shown by pains about the loins, hips, and pubes. On March 14th, she again became my patient for what was stated to be pleurisy, ushered in by intense rigors, followed by heat and sweating. I found her lying in bed on the left side, breathing with extreme difficulty, every respiration being accompanied by a cry of pain; she referred her suffering to a space under the left breast extending to the margin of the ribs. On attempting to turn her in bed, she declared the pain and difficulty of breathing to be insufferable. On examining her chest I could not detect any lesion. Her pulse was 100, small, and rather jerking; pressure over the uterus produced intense pain; her bowels had been constipated for two days, and the leucorrhœal discharge was profuse.

℞. Ol. Ricini. f. ℥j c. T. Opii ♏xx. statim.—Opii, gr. j. Antim. Tart. gr. ¼, ter die.

15th. Numerous scybalous masses were brought away by the oil; she had passed a good night, and had perspired copiously; the pain had nearly ceased. Some wine and porter were allowed her, and she became in a few days once more convalescent. On the 30th March, she walked to my house, apparently well, complaining, however, of a dull pain shooting down the left leg, causing her to draw the limb after her in walking; the leucorrhœal discharge, and the symptoms of irritable uterus still continue. She attributed the pain of the leg to sexual intercourse which had produced such excessive pain, that she fainted.

In this case we have an example of a woman whose nervous system has been from the commencement of menstruation always more or less deranged from an irritable state of the uterus, and its attendant, hysteric epilepsy. The abundant leucorrhœal discharge, even during pregnancy, sufficiently attests the deranged state of the uterine functions. On labour commencing, we find this state reacts on the cerebro-spinal system, and a formidable apoplectic fit, attended with complete unconsciousness, is the result. The labour terminates, she commences suckling her child, and forty-eight hours scarcely elapse ere the peculiar set of symptoms indicative of intense uterine irritation supervene. The suppression of the lochiæ and urine, her position in bed, the pulse, state of the tongue, skin, and abdominal tenderness, all preceded by rigors, would appear to indicate that much dreaded disease, puerperal peritonitis; but the incoherent screams, the wild and unmeaning expression of countenance, the tossing of her arms, and the constant shifting of her position in bed, as well as the crying out, on the mere approach of the hand to the abdomen, and the pain occurring in paroxysms, pointed out, on the other hand, the existence of excessive irritation, and unmasked the true character of the disease. The result of the treatment fully justified the diagnosis. But scarcely had a fortnight elapsed from the time of her convalescence, when, after two days' constipation, rigors again came on, the uterine discharge then ceases, and all the general symptoms of an intense pleurisy appear. Again, a comparison of the symptoms presented by the patient, and an examination of the chest by auscultation, develope the nature of the ailment, and the woman is again relieved. She walks about, performs her ordinary domestic duties, still having severe lumbar and pelvic pains, when a pseudo-paralysis of the left leg, following a direct irritation being applied to the uterus, makes its appearance, all pointing out the internal irritable condition of that organ, and justifying our regarding it as the predisposing cause of the various attacks suffered by this patient after parturition.

I am quite aware that cases of this kind would be by many regarded as essentially arising from intestinal irritation, and would consequently be classed with those interesting cases of head affection described by Dr. M. Hall, as produced by that cause, and so frequently occurring in the puerperal stools. By others, again, these maladies would be, and have been, traced to a strictly constitutional origin. But with regard to the first view, viz., that puerperal neuralgia arises from intestinal irritation, I would remark, that we have equal evidence of the derangement of every function in the body; the secretions of the skin and kidney are in most instances deficient, those of the liver and mucous surfaces are always more or less vitiated, and the bowels, although often constipated, are occasionally acting too energetically, or in some cases are tolerably regular; but the uterine functions are, so far as I have seen, invariably affected, the secretion being either entirely arrested, or becoming very fœtid and viscid, and as one or other of these states always occurs, it is fair to regard them as the essential predisposing causes to the production of the neuralgic state under consideration. Can it be supposed, I would ask, that two days' constipation would alone be adequate to the production of so severe a set of symptoms as we find manifested in the first case detailed in this paper, providing some other important source of irritation had not been exerting its influence upon the patient? Is it, indeed, reasonable to suppose that a slight constipation, a symptom of such frequent occurrence during the puerperal state could develope so serious a train of symptoms, unless some other function was so manifestly imperfect as to produce a generally irritable condition of the nervous system, and thereby rendering the body obnoxious to attacks of disease from exciting causes, which, in the ordinary state, and when free from such depressing influences, would be almost without action upon it? I regard the depressed state of the nervous system produced by the irritable condition of the womb as the predisposing cause of these attacks, and any slightly deranged function, whether of the skin, liver, or bowels, will act as an exciting cause upon such a deranged state of the system, and develope in full force the severe neuralgic attacks under consideration.

With regard to the diagnosis of these neuralgic affections, the comparison of the different symptoms presented by the patient will generally be the best guide of the practitioner. Every symptom of genuine peritonitis may be present in these attacks, but not at the same time; again, the accession of the pain in paroxysms, the screaming out, *ere the hand is in contact with the abdomen*, or when it has barely touched the parietes; the fact of our often being able to apply considerable pressure without pain when the patient's attention is arrested by a sudden exclamation, an abrupt query, or her infant's cries, will generally enable the physician to detect the true nature of the disease. The excessive depression of spirits, and often the strange and groundless delusions of the patient, the accession of an occasional hysteric convulsion, will also assist in forming a correct diagnosis, a task at all times of no small difficulty in these cases.—*Braithwaite's Retrospect*, from *Lond. and Edin. Month. Jour. of Med. Sci.*, Jan. 1843, p. 31.

